# Versatile genetic paintbrushes: Brainbow technologies

**DOI:** 10.1002/wdev.166

**Published:** 2014-12-09

**Authors:** Benjamin Richier, Iris Salecker

**Affiliations:** MRC National Institute for Medical Research, Division of Molecular NeurobiologyLondon, UK

## Abstract

Advances in labeling technologies are instrumental to study the developmental mechanisms that control organ formation and function at the cellular level. Until recently, genetic tools relied on the expression of single markers to visualize individual cells or lineages in developing and adult animals. Exploiting the expanding color palette of fluorescent proteins and the power of site-specific recombinases in rearranging DNA fragments, the development of Brainbow strategies in mice made it possible to stochastically label many cells in different colors within the same sample. Over the past years, these pioneering approaches have been adapted for other experimental model organisms, including *Drosophila melanogaster*, zebrafish, and chicken. Balancing the distinct requirements of single cell and clonal analyses, adjustments were made that both enhance and expand the functionality of these tools. Multicolor cell labeling techniques have been successfully applied in studies analyzing the cellular components of neural circuits and other tissues, and the compositions and interactions of lineages. While being continuously refined, Brainbow technologies have thus found a firm place in the genetic toolboxes of developmental and neurobiologists.

## Introduction

**Figure 1:**
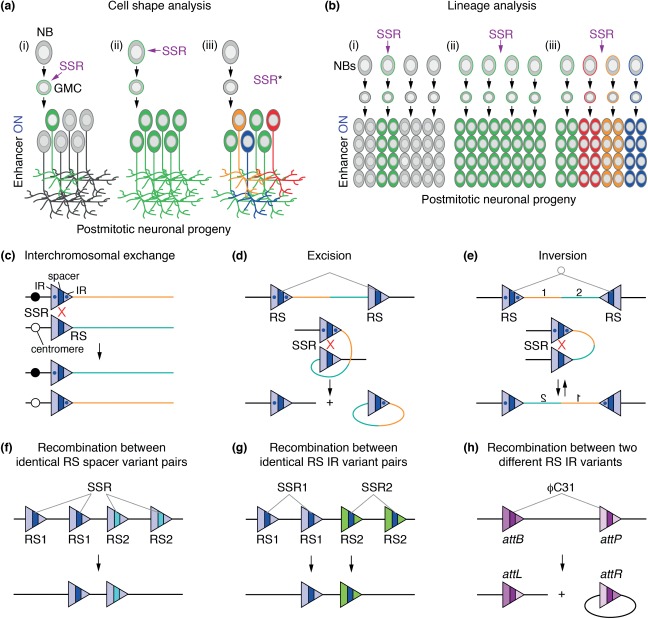
Brainbow techniques take advantage of DNA rearrangements mediated by site-specific recombinases. (a, b) Two aims of multicolor labeling approaches are illustrated in the developing *Drosophila* nervous system, but also apply to other cell types and tissues. Neural stem cell equivalents, the neuroblasts (NBs), self-renew and produce ganglion mother cells (GMCs). These divide to produce postmitotic neuronal progeny, which can be visualized with specific enhancers (ON) driving the expression of reporters. (a) Anatomical studies of overlapping neuron branches require sparse labeling of individual cells. In mosaic approaches, single (i) or lineage-related cells (ii) can be visualized, if recombination events by site-specific recombinases (SSR) are triggered in GMCs or NBs, respectively. Unlike monochrome reporters (ii), sparse multicolor labeling enables the tracing of several neurons in the same sample (iii), even if they are lineage-related or targeted by the same enhancer. SSR*, multicolor labeling can be achieved by recombinase activity in precursors or progeny. (b) Lineage analyses require comprehensive labeling of entire sets of progeny. Activity of SSRs in one precursor allows the labeling of a single clone (i). If a single reporter is used, progeny of multiple lineages can no longer be discerned (ii). Multicolor labeling makes it possible to study several lineages in the same sample (iii). Prior to enhancer activation, FPs are indicated as colored outlines of cells; full expression is indicated by filled cells. (c–h) Cre or FLP SSRs catalyze specific recombination events between pairs of target recombination sites (RS). Each RS consists of two inverted repeats (IR) and a spacer, determining RS directionality. (c) Two RS sites with the same orientation positioned *in trans* trigger the exchange of sequences between homologous chromosome arms. This configuration is used for mosaic approaches, such as MARCM.^63^ (d) SSRs mediate excision of DNA fragments between RS pairs with the same orientation and positioned *in cis*. (e) SSRs catalyze reversible inversions of DNA fragments located between RS pairs with opposite orientations. (f) SSRs mediate recombination between identical pairs of heterospecific site variants that differ in the spacer sequence (blue and cyan). (g) SSR variants are specific for target site pairs with distinct IR sequences (light blue, green). (h) ϕC31 mediates irreversible recombination events between *attB* and *attP* sites, characterized by distinct imperfect IR sequences, to generate new *attL* and *attR* sites.

To address fundamental questions in developmental biology, regardless of the organ, tissue, or model organism, it is essential to visualize cell types of interest. This must be accomplished, as cells divide, migrate, or acquire their mature shapes during normal development and upon functional perturbations. Similarly, detailed information about the morphology and connectivity of neurons within neural circuits is a prerequisite for neurobiological studies aiming at understanding brain function. Ideally in morphological studies, individual cells within genetically defined populations are labeled sparsely within a sample (Figure 1(a)). The targeting of cells critically depends on the enhancers used to drive expression of a visible marker. These may be active in a given cell subtype, but are rarely specific to single cells. Mosaic approaches, combined with cell type-specific enhancers, have thus become instrumental to facilitate single cell labeling.^1^ However, a drawback is that surrounding cells are generally not visible. Consequently, many independent samples are required to assemble a likely incomplete picture of the environment and occurring cell–cell interactions. To assess lineages, one can take advantage of the fact that progeny continue to express the same stable inheritable marker as the precursor, from which they are derived (Figure 1(b)). However, this may limit anatomical studies if a single reporter is used and cell morphology can no longer be unambiguously determined. This can occur when cells form clusters, are born in the same narrow time window or develop extensive overlapping processes. Moreover, to uncover relative contributions of cell lineages, growth rates, or competitive interactions, the ability to track the coordinated behavior of multiple independent clones in the same sample is central. This is particularly beneficial for studies focusing on organ morphogenesis, where comprehensive labeling of proliferating precursors and their offspring is preferred. Labeling with multiple markers thus offers a clear way forward to simultaneously visualize numerous individual cells or complete lineages in the same sample with high resolution (Figure 1(a) and (b)).

Because of the enormous complexity of neuronal shapes and connections in the brain, it was perhaps not unexpected that the strongest need for a genetic multicolor labeling tool was felt by neurobiologists. In 2007, Livet et al.^2^ pioneered a landmark technique to label neurons in a mosaic of many different colors by stochastic and combinatorial expression of a restricted set of fluorescent proteins (FPs) in the mouse brain. This approach was named ‘Brainbow’ because of its primary purpose to map neuronal connectivity. However, it quickly became clear that this technology was also essential for studies in other tissues and model organisms. Thus, over the past years, Brainbow approaches have steadily evolved to circumvent initial limitations and to extend their functionality in response to the requirements of different fields. This review introduces the key genetic building blocks employed by multicolor labeling methods. It then provides a guide to currently available technologies in different model organisms for studies in the nervous system and beyond.

## The Building Blocks

### FPs as Imaging Probes

Brainbow approaches (Table1) would not have been possible without the development of spectrally separable FPs as genetically encoded visible markers and the progress in imaging technologies. The founding member of FPs, Green fluorescent protein (GFP), was discovered in jellyfish *Aequorea victoria* as partner of the bioluminescent protein Aequorin in 1962.^19^ It was successfully cloned for transgenic expression in prokaryotic and eukaryotic cells in 1994.^20^ GFP can be excited with blue light to emit a green fluorescence signal. The subsequent isolation of naturally occurring FPs from other hydrozoan species,^21^ anthozoan corals, such as *Clavularia sp*.,^22^ *Discosoma sp*.^23^ and *Fungia concinna*,^24^,^25^ or the sea anemone *Entacmaea quadricolor*,^26^ as well as systematic bioengineering approaches expanded the color palette ranging from blue to red (Table2). For instance, jellyfish-derived GFP was modified to create blue, cyan, and yellow FPs,^31^–^33^,^37^ while *Discosoma*-derived DsRed was used to generate orange, red, and far-red variants.^41^,^39^ Furthermore, because natural FPs have the basic property of forming dimers or tetramers, mutations were introduced that produce functional monomeric versions.^40^ Additional amino acid changes increased the brightness, maturation rate and photostability, or decreased pH and temperature sensitivity.^39^,^50^

**Table 1:** Summary of Described Multicolor Labeling Techniques

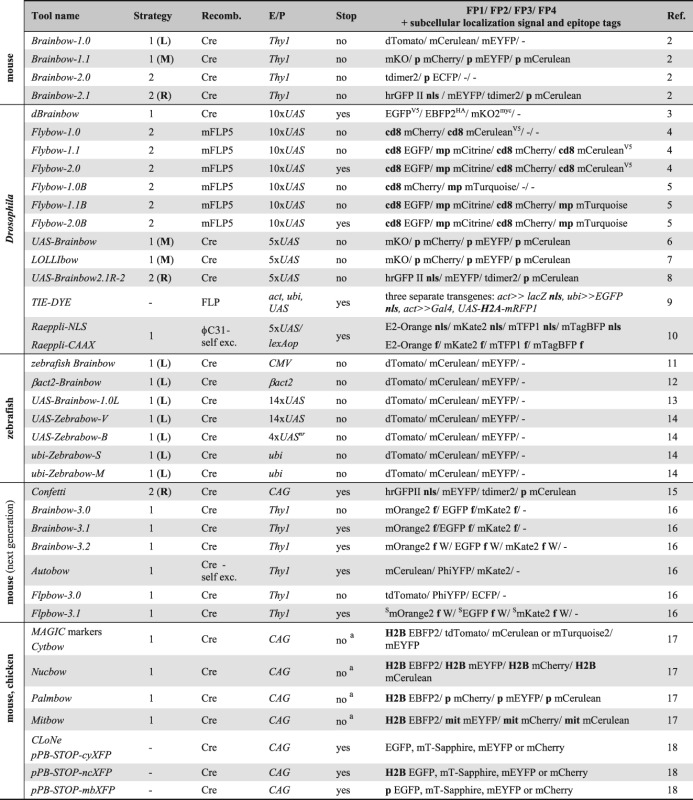

**Table 2:** Fluorescent Proteins and Subcellular Tags Used by Multicolor Labeling Techniques

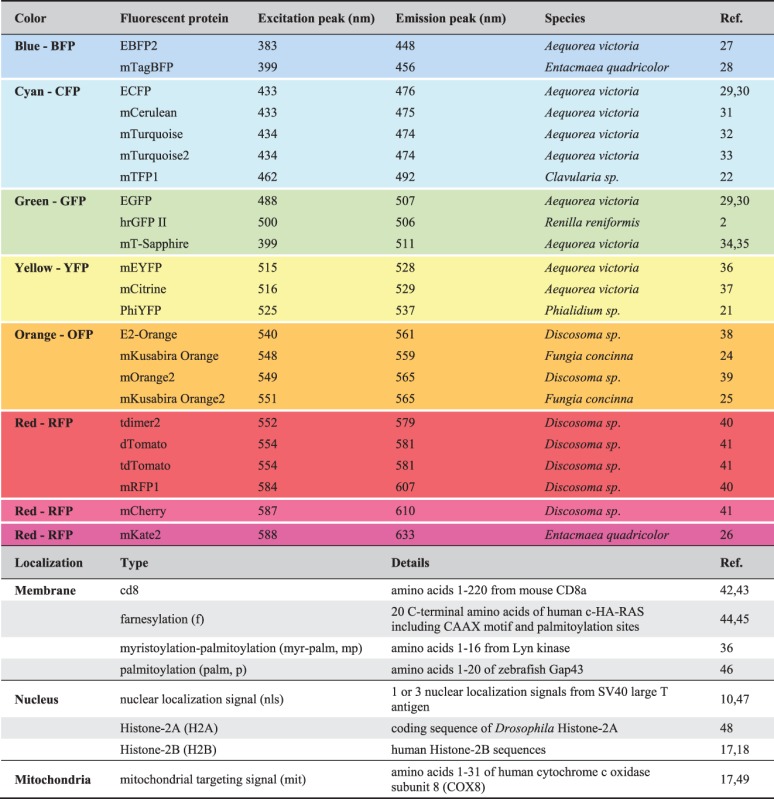

FP emission signals can be collected directly to visualize expression in living or fixed cells by confocal laser scanning microscopes that are equipped with different laser lines and highly sensitive detection devices able to perform spectral separations of narrow wavelength bands. Some FPs are also compatible with multiphoton excitation methods to facilitate imaging of thick samples.^51^ Tissue-clearing technologies, such as Sca*l*e^52^ and CLARITY^53^ further increase image resolution. Moreover, because proteins from jellyfish, coral, and sea anemone species are antigenically distinct and FPs are easily epitope-tagged, expression can also be detected by immunofluorescence labeling using primary antibodies directed either against the FP variant or tags.^3^,^4^,^16^ In all cases, imaging software is used to assign specific colors (e.g., green, yellow, red, or blue) to signals collected in different channels. These can—but do not need to—match the emission spectra of FPs or fluorophore-coupled secondary antibodies. Mixed colors are achieved by the overlay of images acquired in each channel.

Unmodified FPs accumulate in the somatic cytoplasm and only partially spread into cellular processes. For studies in the nervous system, where extensive dendritic and axonal arbors have to be visualized, it is thus helpful to use membrane anchors. In Brainbow technologies, these include a mouse Cd8a sequence,^4^,^42^,^43^ a farnesylation signal from Ras,^10^,^16^,^45^,^54^ a myristoylation-palmitoylation (myr-palm) sequence from Lyn kinase^4^,^36^ and a palmitoylation sequence from Gap43.^2^,^6^–^8^,^17^,^46^ The GRASP approach (GFP reconstitution across synaptic partners) employs a truncated version of the human T cell protein Cd4 as a membrane-tether of GFP fragments in worms and flies.^55^,^56^ This anchor has also successfully been used to generate membrane-bound versions of tdTomato,^57^ and thus could serve as a valuable alternative in future Brainbow constructs. Additionally, FPs are targeted to the nucleus using a nuclear localization signal or Histone-2A and 2B sequences.^2^,^8^–^10^,^15^,^17^,^47^,^48^ Finally, subcellular targeting of FPs to mitochondria is achieved with a sequence from the human cytochrome c oxidase subunit 8 (COX8)^17^,^49^ (Table2).

### Control of Transgene Expression

Reporter expression is achieved by tissue- or cell type-specific enhancer elements. Multicolor cell labeling approaches in vertebrate model organisms rely on the direct transcriptional activation by neuron-specific or ubiquitous enhancers (Table1). By contrast, *Drosophila* transgenes benefit from the flexibility offered by binary expression systems. In particular, the Gal4/*UAS* system has become a corner stone of genetic studies in flies.^58^ In this approach, the yeast-derived transcription factor Gal4, when expressed under the control of a specific enhancer, binds tandem *Upstream Activating Sequences* (*UAS*), which activate transcription of genes including those encoding visible markers. In *Drosophila*, a large number of Gal4 driver lines has been generated that show specific activities in different tissues and cell types during development and in adults. These can be enhancer trap insertions, as well as lines, in which Gal4 is expressed under the control of defined enhancer fragments.^59^–^62^ Further spatiotemporal control can be achieved by the Gal4 repressor Gal80.^63^ When two different enhancers with activities in partially overlapping cell populations are used, expression of Gal4 is solely possible in the subgroup of cells that does not express Gal80. Moreover, a temperature-sensitive Gal80 variant can control expression in developmental time windows.^64^ Other binary expression systems such as LexA/*lexAop* and QF/*QUAS* have recently been introduced as complementary approaches.^65^–^67^ These tools can be combined to enable independent manipulation of different cell types or to refine expression to a few or even single cell types in intersectional strategies.

### Mosaic Expression with Site-Specific Recombinases

To facilitate controlled mosaic expression of visible markers in cells of interest, Brainbow technologies rely on a third set of genetic tools—the site-specific DNA recombinases. These mediate recombination between specific short DNA sequences by catalyzing strand cleavage, exchange and ligation.^68^–^70^ They are grouped into tyrosine or serine recombinases depending on the amino acid required for the catalytic reaction.^71^ Multicolor cell labeling approaches utilize most commonly the tyrosine recombinases Cre and FLP (Table3). Cre is derived from the bacteriophage P1 and specifically recognizes *loxP* [*locus of cross-over (X) in P1*] sites, while FLP recombinases were isolated from *Saccharomyces cerevisiae* and bind to *FRT* (*FLP recombinase target* ) sites. Both minimal *lox* and *FRT* sites consist of two 13-bp inverted repeats.^69^,^72^,^73^ These flank an asymmetric 8-bp spacer sequence, which confers directionality to the sites.^74^ The relative positions and orientation of *lox* or *FRT* pairs determine the outcome of recombination events. When two identical sites are located on homologous chromosomes, interchromosomal recombination events can be triggered during cell divisions. In *Drosophila*, *FRT* sites positioned close to centromeres have been particularly useful for generating somatic clones that are homozygous for genes of interest, while all other cells in the animal are heterozygous^70^ (Figure 1(c)). Intrachromosomal recombination events can lead either to excision of a DNA sequence positioned between *lox* or *FRT* pairs, when they have the same orientation, or inversion, when they have the opposite orientation^69^,^86^ (Figure 1(d) and (e)). Excisions constitute irreversible events because only one functional site remains. By contrast, inversions are reversible because two functional sites are recreated.

**Table 3:** Site-Specific Recombination Systems Used by Multicolor Labeling Techniques

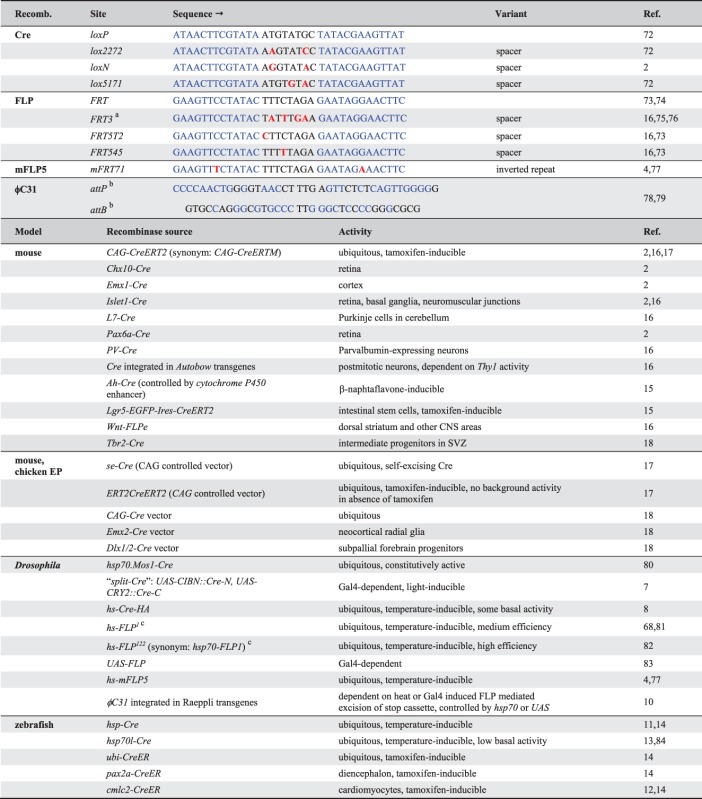

While Cre can recognize several *lox* sites, which differ in the spacer sequence (e.g., *loxP*, l*ox2272, lox5171*, and *loxN*),^2^,^87^ they solely mediate recombination events between identical spacer variant pairs. Similarly, FLP can distinguish *FRT* spacer variants (e.g., *FRT3*, *FRT5T2,* and *FRT545*^16^,^73^,^75^,^76^ (Figure 1(f)). In addition, a FLP variant, mFLP5, shows high specificity for a modified site, *mFRT71*, characterized by sequence changes in the inverted repeats, and little to no cross-reactivity with canonical *FRT* sites^4^,^77^ (Figure 1(g)). Finally, new recombinases from different yeast species—KD, R, B2, and B3—were recently added to the genetic toolbox, which mediate recombination events of four distinct target sites.^85^

Whereas most genetic approaches in vertebrates rely on Cre, this recombinase has found only limited applications in *Drosophila* for two reasons. First, initially generated Cre transgenes in *Drosophila* show constitutive activity that cannot readily be controlled in time or space.^80^ Cre is therefore primarily used for manipulations where efficient excisions in many cells including the germ line are desired (e.g., Ref 88). Second, it shows toxicity in proliferating cells upon persistent over-expression likely due to chromosomal aberrations caused by recombination of pseudo *loxP* sites.^89^ Because the lower efficiency of FLP is suitable for mosaic analysis experiments, this recombinase therefore has become the preferred tool for this type of genetic manipulations in flies.

ϕC31 integrase from the *Streptomyces* bacteriophage belongs to the family of serine recombinases.^78^,^79^ This enzyme catalyzes the unidirectional recombination between bacterial attachment (*attB*) sites, often positioned in plasmids, and phage attachment (*attP)* sites, serving as genomic landing sites. Recombination events are irreversible because the newly generated *attL* (Left) and *attR* (Right) sites are no longer recognized by the integrase (Figure 1(h)). In *Drosophila*, this system is used for controlled genomic integration of DNA sequences,^90^,^91^ cassette exchange,^92^ or fragment excisions.^10^

The regulation of recombinase expression is used to influence the timing and frequency of events (Table3). Importantly, this does not impact on the levels of markers because their expression is under the control of an independent enhancer. To transiently induce high expression in *Drosophila* or zebrafish, FLP and Cre recombinases are placed downstream of a heat shock promoter.^14^,^68^,^80^,^82^ In fish and mammals, temporal activation can also be achieved with the help of inducible Cre transgenes (e.g., CreER and the more sensitive variant CreERT2), in which the recombinase has been fused to a modified ligand-binding domain of the human estrogen receptor.^87^ This receptor is insensitive to endogenous estrogens but can be activated by a synthetic ligand, 4-OH tamoxifen. Cre is retained in the cytosol, and becomes functional after binding of tamoxifen and localization to the nucleus.^87^ For approaches that rely on irreversible excisions, spatiotemporal control of Cre or inducible Cre can also be achieved by tissue-specific enhancer fragments.^87^ To achieve tissue or cell-type specificity, *Drosophila* multicolor labeling techniques tend to control the expression of Brainbow transgenes, whereas tools designed for mice frequently restrict recombinase expression.

## Assembly into Genetic Multicolor Labeling Tools

### Brainbow Blueprints

**Figure 2:**
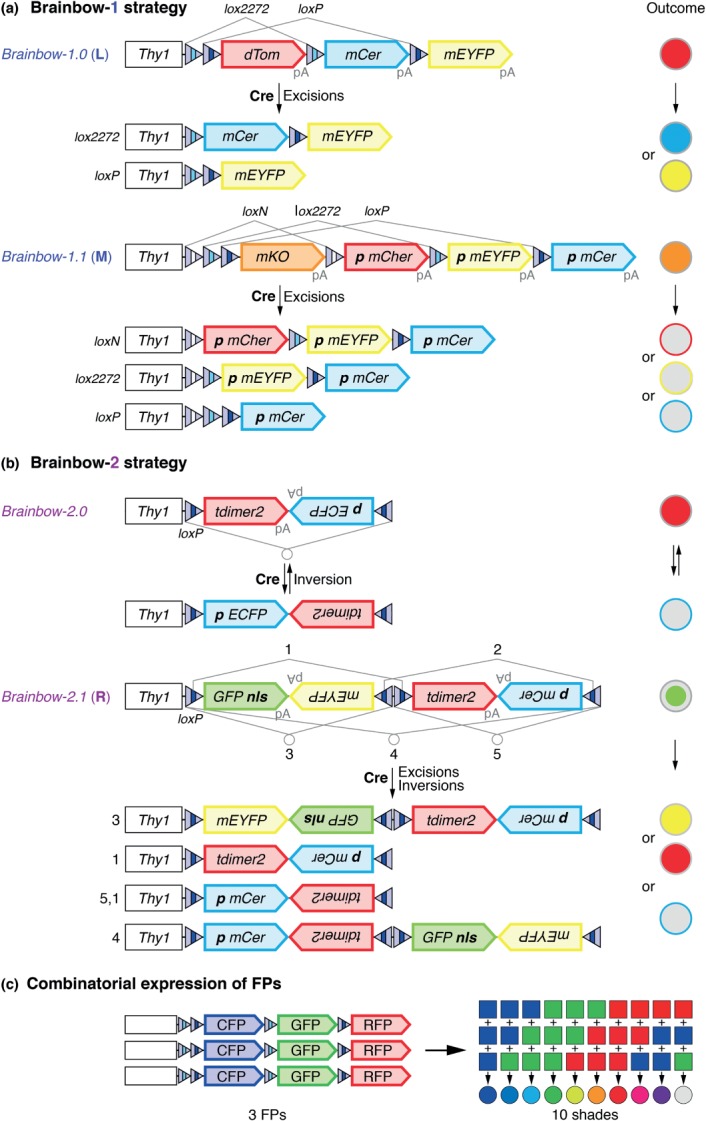
Principles of mouse Brainbow blueprints. (a) Brainbow-1 strategy transgenes (blue) build on the ability of Cre to mediate excisions between heterospecific *lox* pairs orientated in the same direction. In *Brainbow-1.0 (L)*, dTomato (dTom) is expressed by default. Cre catalyzes recombination events between *lox2272* or *loxP* pairs, resulting in the stochastic expression of mCerulean (mCer) or mEYFP, respectively. In *Brainbow-1.1 (M)*, the default marker is mKusabira Orange (mKO). Cre mediates recombination between *loxN*, *lox2272,* and *loxP* pairs, allowing the expression of mCherry (mCher), mEYFP or mCerulean. A palmitoylation signal (*p*) targets these FPs to the membrane. pA, polyadenylation signals. (b) Brainbow-2 strategy transgenes (purple) use the ability of Cre to mediate inversions and excisions between *loxP* sites oriented in the opposite and the same direction, respectively. *Brainbow-2.0* consists of one invertible cassette. tdimer2 is expressed by default. Cre triggers reversible inversions between *loxP* sites, inducing expression of palmitoylated ECFP. *Brainbow-2.1 (R)* consists of two invertible cassettes. Nuclear (nls) GFP is expressed by default. Cre-mediated inversions and excisions between *loxP* pairs allow expression of mEYFP, tdimer2 or palmitoylated mCerulean. All transgenes are under the control of the nervous system specific *Thy1* enhancer. (c) Combinatorial expression of blue, green, and red FPs from three transgene copies increases the color palette from 3 to 10 hues. References for transgenes are provided in Table1.

Exploiting the expanding FP color palette and site-specific recombination technologies, multicolor labeling was first achieved by the Brainbow system devised by Livet et al. for mice.^2^ This creative approach takes advantage of the Cre-*lox* system to stochastically drive the expression of one of three or four FPs from a single transgene in genetically defined cell populations. Brainbow transgenes follow two principles. The Brainbow-1 strategy relies on Cre-mediated excision of DNA fragments using heterospecific *lox* sites (Figure 2(a)). In *Brainbow-1.0* and -*1.1* transgenes, three *lox* pairs (*loxN, lox2272*, and *loxP*) are astutely positioned in the same orientation adjacent to three or four linearly arranged FP-encoding cDNAs. These are each followed by *polyA* termination sequences to prevent transcriptional read-through. The FP located closest to the promoter is expressed by default. Upon Cre activation, site-specific recombination between identical *lox* pairs causes the excision of one, two, or three FP sequences. Consequently, new FPs are randomly positioned closest to the promoter. This leads to the stable, mutually exclusive expression of one of three or four FPs per cell in a tissue. By contrast, the Brainbow-2 strategy makes use of inversion and excision events between a single type of recombination site, *loxP* (Figure 2(b)). The coding sequences of two FPs are arranged in opposite orientations in an invertible cassette flanked by inward-facing *loxP* sites. *Brainbow-2.0* contains one such cassette, and Cre-mediated inversion results in the differential expression of two markers. *Brainbow-2.1* transgenes consist of two adjacent cassettes. Recombination of *loxP* pairs in opposite or identical orientation leads to inversion and excision of cassettes, respectively. This results in four color-outcomes. Because inversions are reversible, transient Cre expression is required. Brainbow transgenes are controlled by the *Thy-1* enhancer to activate expression in neurons or glia, while recombination events are mediated by ubiquitous or tissue-specific Cre transgenes.

Color diversity can be in principle increased by adding more FPs with different emission spectra or epitope-tags to the constructs. However, this strategy is limited by the spectral emission signals of available FPs that confocal microscope detectors can realistically separate with sufficient brightness. Brainbow transgenes therefore use an alternative approach, the combinatorial expression of three or four FPs in two or more copies to increase the number of hues in a sample^2^ (Figure 2(c)). Tandem integration of constructs into the mouse genome after injection into oocytes allows independent combinatorial expression of markers from multiple transgene copies. Depending on the number of transgenes present, cells can be labeled in =100 hues and individually traced by distinct color profiles using sophisticated image processing and analysis software.

### Adjustments for Use in Flies, Zebrafish, Mice, and Chicken

The two original Brainbow strategies served as blueprints for the subsequent development of multicolor labeling technologies in *Drosophila* and vertebrate model organisms. Adjustments include specific adaptations for each animal species, as well as optimizations to overcome initial drawbacks and to increase the versatility of approaches.

#### Drosophila Multicolor Cell Labeling Approaches

Naturally, because of their alluring genetics, Brainbow technologies found their way into the toolbox of Drosophilists. *dBrainbow*^3^ and *Flybow*^4^ transgenes made a start on adapting the original strategies for use in flies. To take advantage of the increasing number of available Gal4 lines for controlling expression in any genetically accessible cell subpopulation and tissue of interest, both methods rely on *UAS* activated transcription. To boost the expression levels of reporter genes, constructs use 10 instead of 5 *UAS* repeats. Moreover, to avoid position effects, constructs are inserted into the genome by ϕC31-mediated integration into genomic *attP* landing sites that show a high level of expression in the presence of Gal4 and no residual expression in its absence.

**Figure 3:**
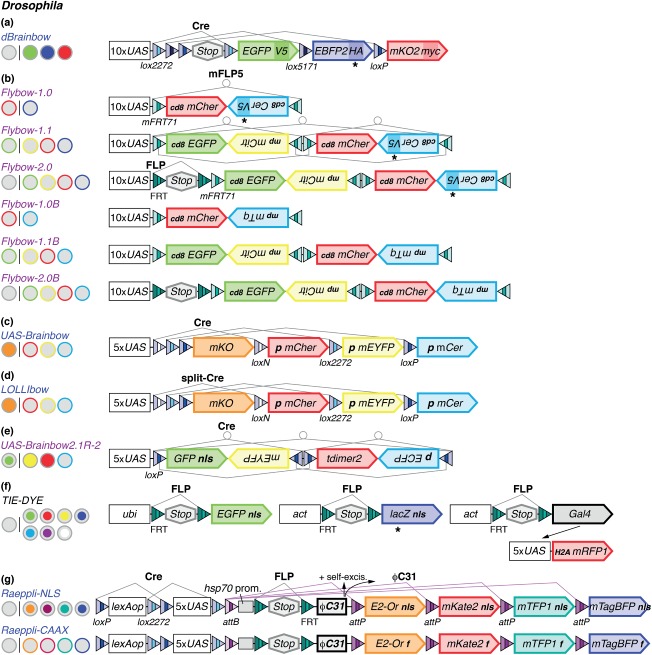
Multicolor labeling tools in *Drosophila.* Transgenes following the excision-based Brainbow-1 strategy are highlighted in blue. Transgenes modeled on the inversion/excision-based Brainbow-2 strategy are shown in purple. Constructs are downstream of upstream activation sequences (*UAS*). (a) In *dBrainbow*, a stop cassette prevents marker expression prior to Cre activation. FPs are detected with three epitope-tags. Native fluorescence signals can be collected for EGFP and mKusabira Orange2 (mKO2); EBFP2 requires detection by immunolabeling (asterisk). (b) In *Flybow*, FPs are membrane-tethered using cd8 or myristoylation-palmitoylation (mp) sequences. *Flybow B* transgenes use mTurquoise (mTq) instead of V5-tagged mCerulean (mCer), which requires immunodetection (asterisk). *Flybow-1.0*, *1.1*, *1.0B,* and *1.1B* transgenes show default expression of mCherry (mCher) or EGFP. *Flybow-2.0* and *2.0B* require FLP-mediated excision of a *FRT*-site flanked stop cassette. Recombination events between *mFRT71* sites are triggered by mFLP5. (c-e) *UAS-Brainbow*, *LOLLIbow,* and *UAS-Brainbow2.1R-2*, are derived from the mouse Brainbow transgenes M and R. Recombination events are mediated by Cre. *LOLLIbow* relies on photo-activated split-Cre. p, palmitoylation signal. (f) In *TIE-DYE*, FLP mediates the excision of stop cassettes in three separate transgenes controlled by *ubiquitin* (*ubi*) or *actin* (*act*) enhancers. Gal4 leads to expression of mRFP1. *lacZ* requires detection with an antibody against βGal (asterisk). Seven color outcomes are possible for the combination of these markers, targeted by a nuclear localization signal (nls) or Histone-2A (H2A). (g) *Raeppli* transgenes are downstream of *lexAop* or *UAS*. Cre-mediated excision converts transgenes into exclusively Gal4 or LexA controlled constructs. FLP-mediated excision of a *FRT*-flanked stop cassette, enables ϕC31 transcription. Integrase expression is controlled by the full heat shock protein 70 (hsp70) promoter or *UAS*. This leads to recombination between the *attB* site and one of the four *attP* sites preceding each FP and to integrase self-excision. E2-Or, E2-Orange. FPs label cell nuclei in *Raeppli-NLS*, and cell membranes using a farnesylation (f) signal in *Raeppli-CAAX*. References for transgenes are provided in Table1.

*dBrainbow*^3^ is modeled on the Brainbow-1 strategy and uses Cre-mediated recombination of heterospecific *lox* sites (Figure 3(a)). A transcriptional stop cassette precedes the series of three FP-encoding sequences to ensure that cells are solely labeled upon Cre expression. Moreover, each FP is tagged with a different epitope (V5, HA, and myc), which can be detected by immunohistochemistry. This helps to boost labeling intensities when endogenous fluorescence signals are inherently low or quenched during fixation of tissues. By contrast, Flybow transgenes^4^ are based on the Brainbow-2 strategy (Figure 3(b)). To bypass the limitations of Cre in flies, *Flybow* uses the mFLP5-*mFRT71* system^77^ as an orthogonal tool that can be combined with the canoncial FLP-*FRT* system. mFLP5 is controlled by the heat-shock promoter. Transient exposure to heat induces the expression of mFLP5, which mediates inversions and excisions of cassettes flanked by *mFRT71* sites. Moreover, to facilitate complete labeling of neurites, all FPs are membrane-tethered.^43^,^36^ Similar to mouse *Brainbow-2.0* and -*2.1* transgenes, *Flybow-1.0* and -*1.1* constructs consist of one and two cassettes, respectively. In *Flybow-2.0*, an additional transcriptional stop cassette flanked by *FRT* sites in the same orientation precedes the invertible cassettes to eliminate default marker expression. The stop cassette is excised after induction of the canonical FLP recombinase. Transient FLP expression facilitates both sparse labeling and increases the color diversity because all four FPs can be used for tracing. Because *Flybow-2.0* relies on both FLP and mFLP5, it additionally can serve as an intersectional tool to refine expression, when FLP expression is controlled by a different cell-specific enhancer. The initial set of *Flybow* constructs uses an epitope-tagged cyan FP mCerulean variant,^31^ which requires immunodetection because of its weak native emission in flies. To bypass the need for immunolabeling and to enable live imaging of endogenous fluorescence signals in all four channels, in a second set of transgenes (*Flybow-1.0B*, *1.1B* and *2.0B*)^5^ cd8-tethered mCerulean-V5 was replaced by the brighter myr-palm anchored mTurquoise.^32^

Three subsequent *Drosophila* multicolor cell labeling methods take advantage of original mouse *Brainbow-1* and -*2* constructs. In *UAS-Brainbow* (Figure 3(c))^6^ and *LOLLIbow* (live imaging optimized multicolor labeling by light-inducible Brainbow; Figure 3(d)),^7^ the mouse *Brainbow-1.1 (M)* cassette was inserted into different *UAS* vectors. Similarly, in *UAS-Brainbow2.1R-2* (Figure 3(e)),^8^ the mouse *Brainbow-2.1 (R)* fragment containing two invertible cassettes was transferred into a *UAS* vector. The constitutively-active *hsp70-Mos1-Cre* (Ref 80) and a new heat shock-inducible *hs-Cre-HA* line^8^ are used in conjunction with *UAS-Brainbow* and *UAS-Brainbow2.1R-2* transgenes, respectively. By contrast in *LOLLIbow*, recombination events are controlled by a photo-inducible split-Cre version.^7^ The N- and C-terminal fragments of Cre were fused with two plant proteins—a truncated version of CIB1 (CIBN) and nuclear targeted Cryptochrome 2 (CRY2)—and subcloned into *UAS* vectors. Gal4 activates the simultaneous expression of these chimeric proteins while brief exposure to blue light induces their dimerization to reconstitute a functional enzyme.

Two other additions to the toolkit, *TIE-DYE*^9^ and *Raeppli,*^10^ have been designed to support whole-tissue multicolor labeling by increasing the recombination efficiency. Unlike the other approaches, *TIE-DYE* (three independent excisions dye) does not rely on a single but a combination of four separate, previously generated transgenes^9^ (Figure 3(f)). Upon heat shock, FLP induces the expression of nuclear GFP, β-Galactosidase or Gal4 by stochastically excising FLP-out stop cassettes that are positioned between the widely active enhancers *actin* or *ubiquitin* and the reporters. Gal4 in turn activates expression of nuclear RFP from a fourth *UAS* controlled transgene. When visualizing *lacZ* by immunolabeling with a secondary antibody coupled to a far-red fluorophore, clones can be labeled in up to seven hues, because combinations of several excision events can occur in each cell. Moreover, in conjunction with *UAS*-based knockdown or over-expression transgenes, effects of genetic manipulations on subsets of clones that co-express RFP can be compared with control clones that express GFP and/or β-Galactosidase but not RFP. *Raeppli* (named after the Basel carnival confetti)^10^ makes use of all three recombination systems in a single versatile transgene (Figure 3(g). Transgenes are under the dual control of *lexAop* and five *UAS* repeats, which are flanked by heterospecific *lox* pairs, leaving all options open for genetic manipulations by two independent binary systems. Moreover, Cre can be used to catalyze excision events that produce stable strains with restricted *UAS* or *lexAop* controlled transgenes in the same genetic locus. Because insertions are influenced by their chromosomal positions, this trick ensures that expression levels remain identical, since no additional injections are required to generate separate lines. *Raeppli* follows the Brainbow-1 strategy to control the selection and expression of markers. However, instead of Cre or FLP, ϕC31 catalyzes the excisions. One *attB* site follows the *lexAop* and *UAS* repeats, while *attP* recombination sites each precede the sequences of four linearly arranged FPs. These are either nuclear or targeted to the membrane by a farnesylation signal. Importantly, the transgenes include the ϕ*C31* coding sequence, which has been placed downstream of a full *heat shock protein 70* (*hsp70*) promoter. A *FRT*-flanked stop cassette, positioned between the promoter and the integrase, reduces low-level background activity of ϕC31 while providing means for temporal control. Heat- or Gal4-induced FLP leads to excision of the stop cassette. This enables ϕC31 expression controlled by the activity of the heat shock promoter or *UAS*. The integrase in turn catalyzes the recombination between the *attB* and one of the four *attP* sites, resulting in the stable selection of one FP, as well as self-excision.

Unlike in mice, fly transgenes integrate as single copies into genomic loci. The number of *UAS*-controlled transgenes can be increased by standard genetic crosses. Doubling the transgenes with three or four FPs extends the number of detectable hues to 6 or 10.^3^,^7^,^10^ The addition of further copies in one animal is possible but genetic crosses become increasingly complex due to the limited set of chromosomes. Because *Drosophila* multicolor labeling tools use the Gal4-*UAS* system, they can readily be combined with *UAS*-based RNA interference or over-expression constructs for knockdown and gain-of-function approaches.^93^,^94^ Moreover, Brainbow tools that do not rely on canonical FLP-*FRT* site-specific recombination and involve a small number of transgenes, can also be combined with loss-of-function approaches such as mosaic analysis with a repressible cell marker (MARCM).^63^

#### Adaptations in Zebrafish

**Figure 4:**
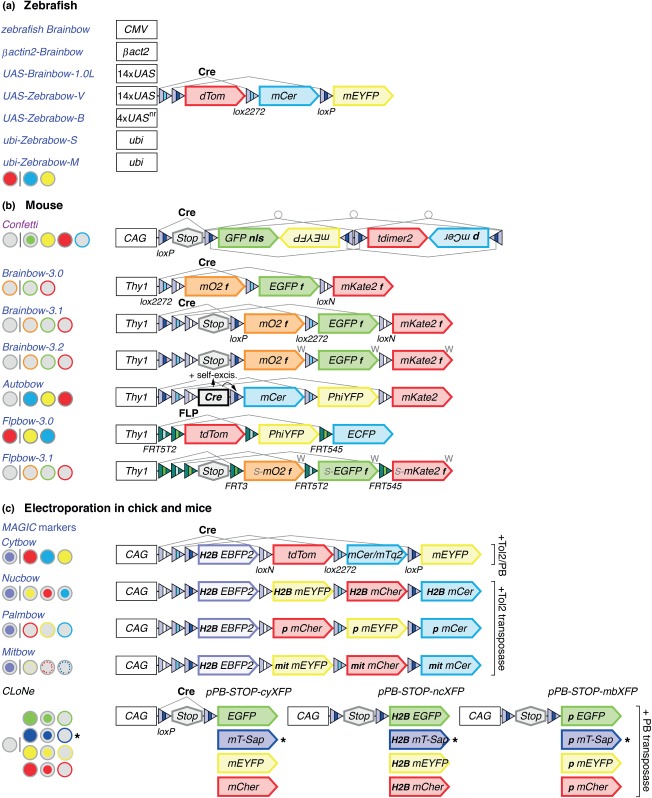
Multicolor labeling tools for use in zebrafish and mouse, as well as for electroporation in mouse and chicken. Constructs following the Brainbow-1 strategy are indicated in blue and constructs based on the Brainbow-2 strategy in purple. (a) In zebrafish, the mouse *Brainbow-1.0L* cassette has been placed downstream of four regulatory elements: the *cytomegalovirus* enhancer (*CMV*), the *βactin2 (βact2*) enhancer, upstream activation sequences (*UAS*), or the ubiquitin (*ubi*) enhancer. *UAS-Zebrabow-B* uses non-repetitive (nr) tandem *UAS* sites. V, variegated; B, broad; S, single; M, multiple. (b) In *Confetti*, a stop cassette precedes the two invertible cassettes of the original mouse *Brainbow-2.1 (R)* transgene. Expression is controlled by *CAG*, the *chicken β-actin promoter with cytomegalovirus* (*CAG*) enhancer. Grey lines only indicate a subset of possible recombination events. *Thy1*-controlled *Brainbow-3.0, 3.1, 3.2* trangenes use farnesylated (f) FPs – mOrange2 (mO2), EGFP, and mKate2. In *Brainbow-3.1* and *3.2,* a stop cassette prevents default FP expression. In *Brainbow-3.2*, a woodchuck hepatitis virus posttranscriptional regulatory element (W) has been placed downstream of each FP. In *Autobow*, *Thy1* controls expression of *lox*-flanked Cre to trigger recombination events and self-excision. In *Flpbow-3.0* and *3.1* transgenes, FLP mediates recombination of spacer variant pairs *FRT3*, *FRT5T2,* and *FRT545*. *Flpbow-3.1* uses a stop cassette and FP fused with a SUMOstar tag (S) for immunodetection. mCer, mCerulean; PhiYFP, Phialidium YFP; tdTom, tandem dimer Tomato. (c) *MAGIC* and *CLoNe* plasmids are transposon-based vectors suitable for electroporation experiments in mouse and chicken. Tol2 or PiggyBac (PB) transposases promote the genomic integration of vectors. Ubiquitous or tissue/cell-type specific Cre is provided by co-injected vectors (chick and mouse) or by expression from genomic insertions (mouse). FP expression is controlled by the *CAG* regulatory element. In *MAGIC* markers, four different FPs are expressed from single vectors. FP are localized in the cytoplasm (*Cytbow*), in nuclei using Histone-2B (H2B) fusions (*Nucbow*), in the membrane using a palmitoylation (p) signal (*Palmbow*) or in mitochondria (mit) using a targeting signal from human *COX8 (Mitbow)*. H2B-EBFP2 is expressed in unrecombined cells. In the *CLoNe* approach, four FPs [EGFP, mT-Sapphire (mT-Sap), mEYFP, and mCherry] are expressed from twelve separate labeling vectors. FPs are either cytoplasmic (cy), or nuclear (nc), and membrane-bound (mb) using H2B or palmitoylation tags, respectively. A stop cassette prevents default expression of markers in the absence of Cre. Stable multicolor labeling is achieved by different random combinations of vector insertions and expression in individual cells. Asterisk indicates that mT-Sapphire was assigned the color blue, although the maximum emission is in the green/yellow range. References for transgenes are provided in Table1.

Multicolor labeling approaches developed for zebrafish utilize the mouse *Brainbow-1.0 (L)* cassette (Figure 4(a)). In *zebrafish Brainbow*, this cassette is positioned downstream of the *cytomegalovirus* (*CMV*) promoter in a commonly used expression vector, that allows transient ubiquitous expression after injection.^11^ *βactin2-Brainbow* goes one step further by using an *actin* enhancer in a stable transgenic line.^12^ In *UAS-Brainbow-1.0L*,^13^ and Zebrabow,^14^ tissue-specific transgene expression is controlled either by 14 *UAS* repeats (*UAS-Brainbow1.0L* with at least 3 insertions and *UAS-Zebrabow-V* with 2 insertions) or four non-repetitive *UAS* sequences (*UAS-Zebrabow-B* with 9–31 copies). In the presence of Gal4, the former results in variegated mosaic expression because repeat sequences are prone to CpG methylation and randomly silenced,^95^ thus rendering this strategy highly suitable for sparse labeling. By contrast, tandem *UAS* sites with four unique sequences are less susceptible to methylation, thus enabling broad cell labeling. Additional Zebrabow transgenes are controlled by the *ubiquitin* enhancer and either contain a single (*ubi-Zebrabow-S*) or multiple (16–32) insertions (*ubi-Zebrabow-M*).^14^ While transgenic lines with a high number of insertions in principle produce many hues, data analysis may be limited by the capacity of image processing software in achieving the necessary resolution of colors. Site-specific recombination events are mediated by Cre, which can be delivered by microinjection of a purified protein. Alternatively, zebrafish transgenics can be crossed with heat shock-inducible Cre or tamoxifen-inducible CreER lines that either are widely expressed or controlled by tissue-specific enhancers.^14^

#### Second and Third Generations of Mouse Brainbow Transgenes

The expression of a default FP in the first generation of mouse Brainbow strategies represents a potential limitation for some applications. Therefore, in Confetti mice,^15^ a *loxP* flanked stop cassette was added upstream of the two invertible cassettes in the *Brainbow-2.1 (R)* transgene. Similar to *dBrainbow*^3^ and *Flybow 2.0,*^4^ this ensures that cells express FPs only after recombinase-mediated excision of this cassette.

Six years after their first publication,^2^ mouse Brainbow transgenes underwent a redesign.^16^ *Brainbow-3.0* transgenes switched to different FPs, mOrange2, EGFP and mKate2, because they are spectrally and antigenically distinct, show less tendency to aggregate and are highly stable upon illumination and after fixation. To evenly label axons and dendrites, the FPs were membrane-tethered using a farnesylation sequence. In *Brainbow-3.1*, a non-fluorescing mutated YFP from the hydrozoan *Phialidium* (PhiYFP) occupies the default position to function as a stop cassette. Importantly, mutated YFP can still be detected by antibody labeling to visualize cells that have not undergone recombination. In *Brainbow-3.2*, a woodchuck hepatitis virus post-transcriptional regulatory element (WPRE) was inserted downstream of each FP sequence to increase protein levels. To reduce the number of required crosses, similar to *Raeppli*, in *Autobow* transgenes a stop cassette containing Cre recombinase cDNA was placed upstream of the linearly arranged FP sequences. When neurons begin to differentiate, the *Thy1* regulatory element leads to expression of Cre. The enzyme subsequently catalyzes recombination events that allow both the expression of FPs and self-excision. While lacking temporal and spatial control, this approach helps to accelerate the analysis of loss-of-function phenotypes because only one transgene needs to be combined with mutant alleles. Finally to serve as orthogonal labeling systems, in two *Flpbow* transgenes, *lox* sites were replaced by incompatible *FRT* spacer variants (*FRT3*, *FRT5T2,* and *FRT545*) to allow FLP-mediated recombination events.

#### Vectors for Embryonic Electroporation in Mouse and Chicken

In mice and chicken, *in utero* and *in ovo* electroporation constitutes a widely used alternative technique to study nervous system development. Injection of plasmids into the brain ventricles, the central canal of the spinal cord or the optic vesicle, and application of an electric current make it possible to perform region-specific transfections of cells during restricted developmental time windows. Although targeted delivery of viral vectors can provide spatial and temporal control,^16^,^96^ applications are limited to neuroanatomical studies. Four recently developed sets of labeling vectors take advantage of the stochastic nature of plasmid integration following electroporation to enable multicolor lineage tracing. To ensure that labels are stably inherited during mitotic divisions, transposon-based vectors are co-electroporated with transposase-expressing plasmids to catalyze genomic integration. Two approaches, *PB IUP* (PiggyBac in utero electroporation)^97^ and *Star Track,*^98^ function independently of Cre and involve the electroporation of mixtures of separate plasmids, each driving expression of a single FP under the control of broadly active or cell-type specific enhancers. In *Star Track*, FPs are either cytoplasmic or targeted to the nucleus. The versatility of these methods is further extended by two additional Cre-dependent toolkits—*MAGIC* (multiaddressable genome-integrative color) markers^17^ and *CLoNe* (clonal labeling of neural progeny).^18^ These enable expression in neural precursors and their offspring, because vectors are under the control of the ubiquitous *CAG* promoter (Figure 4(c)). *MAGIC* and *CLoNe* vectors rely on random genomic integration of a small number of transposon-based vectors (one to three transposons per cell in the case of *MAGIC* markers). Co-injected plasmids in mouse or chick, or stably inserted transgenes in mouse strains serve as sources for Cre. This provides an additional level of spatiotemporal control and specificity because the expression of Cre can be regulated by different enhancer elements. *MAGIC* constructs are based on the Brainbow-1 blueprint, in which stochastic expression of four FPs is obtained by Cre-mediated recombination events in a single vector. The first FP, nuclear EBFP, is expressed by default and followed by tdTomato or mCherry, mCerulean or mTurquoise2 and mEYFP arranged in varying order. In these vectors, FPs are either located in the cytoplasm (*Cytbow*) or targeted to nuclei (*Nucbow*), membranes (*Palmbow*) or mitochondria (*Mitbow*). By contrast, *CLoNe* vectors follow a similar principle as *Drosophila TIE-DYE*, *Star Track* or *PB-IUP*. Random expression of cytoplasmic, nuclear or membrane-bound FPs (EGFP, mT-Sapphire, mEYFP, and mCherry) is achieved by twelve separate vectors. A *loxP*-flanked stop cassette upstream of each FP prevents default expression in the absence of Cre. Importantly, in *Star Track*, *MAGIC* and *CLoNe* approaches, the co-electroporation of vectors with separable subcellular addresses facilitates the identification of clonally related cells by increasing the number of unique marker combinations—e.g., color and marker localization.

## Conclusions

Brainbow technologies are a fascinating habitat for geneticists. The designs in different model organisms clearly inspired each other. The recent progress in molecular cloning techniques facilitated the assembly of genetic building blocks into sophisticated constructs. Moreover, steady technical advances in confocal and multiphoton microscopy made it possible to image differentially labeled cells with increasing ease. Some of the multicolor labeling tools have found their first successful applications in developmental studies in a number of tissues (Box 1) and more will undoubtedly follow. In parallel, new Brainbow methods are still in the making. What features would benefit most urgently from enhancements? Expression constructs generally seem to function well and are suitable for many applications. However, one drawback is still the control over recombination events, which requires most of the adjustments in each experimental situation. Recombination can occur in precursors or postmitotic progeny, independently of cell divisions. For morphological studies, as long as a subset of several cells in a sample has been labeled in different colors and unambiguous tracing is possible, it does not matter whether recombination happens in precursors or their offspring. By contrast, for lineage studies, it is crucial to trigger single recombination events specifically in precursors. In both situations, considering the small number of possible resolvable labels, a highly diverse color outcome is desired: in progeny, as this helps to increase the density of sparse labeling and thus the reconstruction of individual cells, and in precursors to follow many different lineages in parallel. Therefore means need to be found, by which high expression levels of recombinases can be induced transiently, with little delay and at a precise developmental stage. Moreover, recombinase variants could be designed, that are highly efficient at low levels, do not display any background activity and therefore require only brief enhancer activity to induce expression. Finally, similar to the light-inducible *split-Cre* strategy,^7^ inactive recombinase variants could be expressed at high levels from the outset, which are rapidly converted into active variants in response to an external signal applicable in all model organisms.

## Box 1 Brainbow Technologies at Work

**Figure 5:**
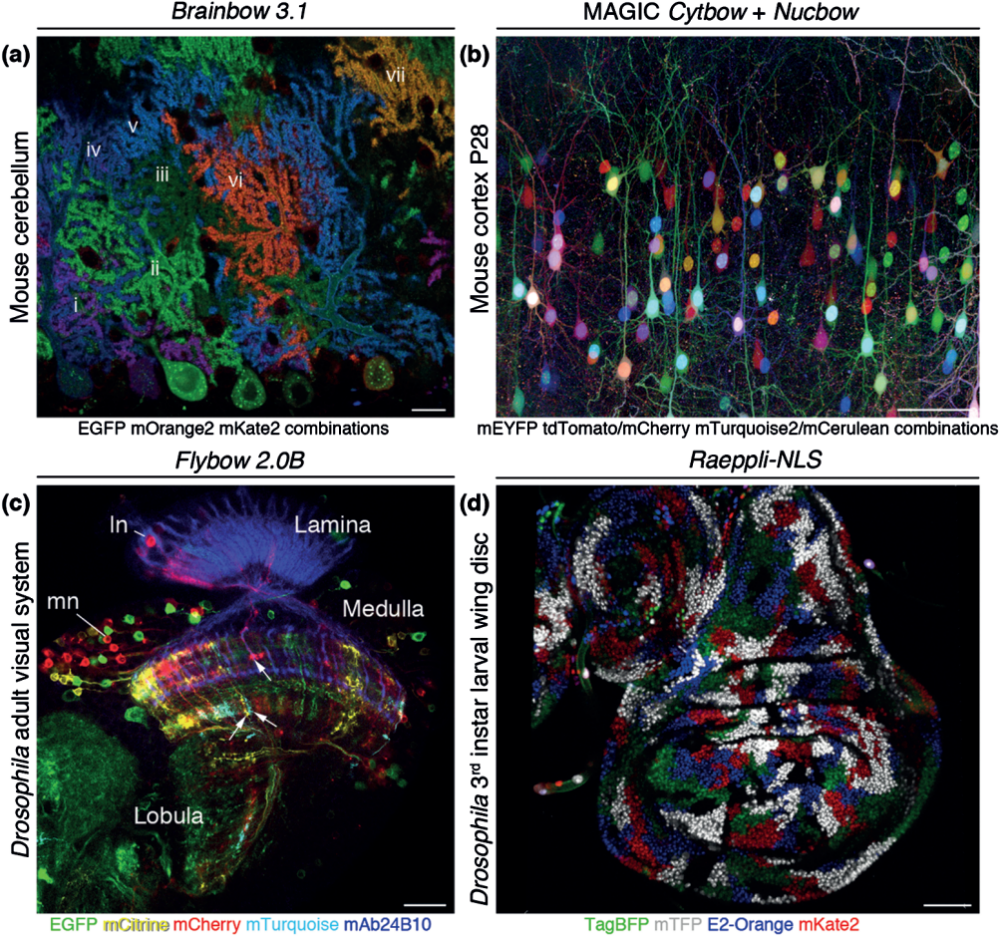
Four examples of Brainbow technologies at work. (a) Purkinje cells in the mouse cerebellum are visualized in seven colors (i–vii) using *Brainbow-3.1* and *L7-Cre* transgenes, as well as antibody amplification. (Reprinted with permission from Ref 16. Copyright 2013 Nature Publishing Group) Scale bar, 20 µm. (b) Pyramidal neurons in the P28 cortex of a *CAG-CreERTM* mouse are labeled by combinations of co-electroporated MAGIC *Cytbow* and *Nucbow* markers at E15. The image was acquired by two-photon microscopy. (Reprinted with permission from Ref 17. Copyright 2014 Elsevier Ltd.) Scale bar, 100 µm. (c) Neurites of lamina and medulla neuron subtypes (ln, mn) in the adult *Drosophila* optic lobe are visualized by endogenous fluorescent protein signals using a *Flybow-2.0B* transgene, activated by *hs-mFLP5* and *NP4151-Gal4*—an enhancer trap insertion into the *Netrin B* locus. The image represents a single optical section. Several neurons (arrowheads) are suitable for tracing in stacks. Photoreceptor axons are visualized by immunolabeling with mAb24B10 (blue). Scale bar, 20 µm. (d) Nuclei of epithelial cell clones in a 3^rd^ instar larval wing disc of *Drosophila* are labeled by four fluorescent proteins using a *Raeppli-NLS* transgene, activated by *tubulin-Gal4* and *UAS-FLP*. This approach facilitates the comprehensive analysis of clones in the entire tissue. (Reprinted with permission from Ref 10. Copyright 2014 The Company of Biologists Ltd.) Scale bar, 50 µm.

Brainbow methods were designed for anatomical and functional studies of genetically accessible cell populations with two main experimental applications in mind: (1) sparse labeling of specific cell types to visualize their morphologies and (2) comprehensive labeling of clonally related cells to track lineages (Figure 5). Consistently, Brainbow transgenes were so far successfully utilized to map known and new neuron subtypes,^8^,^99^ to identify the role of a basic helix-loop-helix transcription factor in axonal projection pattern formation,^100^ and to monitor laminar map assembly^13^ in the visual systems of flies and zebrafish. Furthermore, visualization of single cell shapes in their epithelial environment provided insights into the role of the tyrosine kinase Src42A in embryonic tracheal tube elongation.^6^ In lineage tracing experiments, Brainbow technologies were employed to follow the development of individual embryonic peripheral glial cell subtypes into perineurial, subperineurial, and wrapping glial subtypes associated with third instar larval peripheral nerves in *Drosophila.*^101^ Finally, multicolor clonal analysis discovered the contributions of dominant cardiomyocyte lineages to zebrafish heart morphogenesis,^12^ the role of neutral competition between symmetrically dividing intestinal crypt stem cells^15^ and the origin of stem cells required for corneal epithelial renewal in mice.^102^

Parallel efforts could be dedicated to further extend the functionality of Brainbow approaches by connecting one color outcome with an additional subcellular marker, such as a presynaptic protein, or a specific genetic manipulation. This is to some extent possible with the TIE-DYE approach.^9^ To reduce the number of required transgenes and complexity of genetic crosses, the self-processing 2A peptide sequence^103^ represents a valuable alternative, as it can be placed between two proteins to achieve co-translational cleavage and bicistronic expression. Loulier et al. showed that a dominant-negative form of one molecular determinant and one FP can be co-expressed with the 2A system to report genetic mosaic perturbations in mice.^17^ Similarly in *Drosophila,* LexA, or QF could be linked to one FP and used in conjunction with *lexAop or QUAS*-based knockdown or over-expression transgenes to study the effects of a genetic manipulation on a subset of clones. These possibilities underscore that multicolor cell labeling tools are here to stay and will evolve further. They will continue to unlock doors and provide us with access to our cells of choice, making them visible in bright colors with the strokes of genetic paintbrushes.
